# *N*′-(2,4-Dichloro­benzyl­idene)-4-hy­droxy­benzohydrazide

**DOI:** 10.1107/S1600536810045502

**Published:** 2010-11-13

**Authors:** Hong-Wei Huang

**Affiliations:** a College of Chemistry and Biology Engineering, Yichun University, Yichun 336000, People’s Republic of China

## Abstract

The title hydrazone compound, C_14_H_10_Cl_2_N_2_O_2_, was synthesized by the reaction of 2,4-dichloro­benzaldehyde and 4-hy­droxy­benzohydrazide. The mol­ecule adopts an *E* geometry with respect to the azomethine group and the dihedral angle between the aromatic rings is 7.0 (2)°. In the crystal, mol­ecules are linked through inter­molecular N—H⋯Cl and O—H⋯O hydrogen bonds, forming a three-dimensional network.

## Related literature

For the structures and properties of hydrazones, see: Carvalho *et al.* (2010 ▶); Liu (2010 ▶); Fun *et al.* (2008 ▶); Wang *et al.* (2010 ▶); Singh *et al.* (2009 ▶); Zhu *et al.* (2009 ▶); Vijayakumar *et al.* (2009 ▶); Tameem *et al.* (2010 ▶).
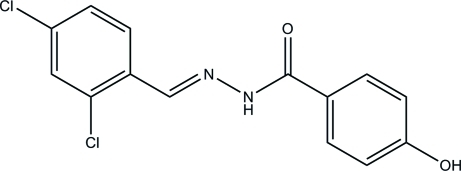


## Experimental

### 

#### Crystal data

C_14_H_10_Cl_2_N_2_O_2_
*M* *_r_* = 309.14
Monoclinic, 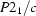

*a* = 7.6687 (11) Å
*b* = 11.9591 (17) Å
*c* = 15.043 (2) Å
β = 103.200 (2)°
*V* = 1343.2 (3) Å^3^
*Z* = 4
Mo *K*α radiation
μ = 0.49 mm^−1^
*T* = 298 K
0.17 × 0.13 × 0.13 mm

#### Data collection

Bruker SMART CCD diffractometer
Absorption correction: multi-scan (*SADABS*; Sheldrick, 1996 ▶) *T*_min_ = 0.922, *T*_max_ = 0.940
6784 measured reflections
2838 independent reflections
2385 reflections with *I* > 2σ(*I*)
*R*_int_ = 0.019

#### Refinement

*R*[*F* ^2^ > 2σ(*F* ^2^)] = 0.041
*wR*(*F* ^2^) = 0.114
*S* = 1.05
2838 reflections
185 parameters
1 restraint
H atoms treated by a mixture of independent and constrained refinement
Δρ_max_ = 0.37 e Å^−3^
Δρ_min_ = −0.58 e Å^−3^

### 

Data collection: *SMART* (Bruker, 1998 ▶); cell refinement: *SAINT* (Bruker, 1998 ▶); data reduction: *SAINT*; program(s) used to solve structure: *SHELXS97* (Sheldrick, 2008 ▶); program(s) used to refine structure: *SHELXL97* (Sheldrick, 2008 ▶); molecular graphics: *SHELXTL* (Sheldrick, 2008 ▶); software used to prepare material for publication: *SHELXTL*.

## Supplementary Material

Crystal structure: contains datablocks global, I. DOI: 10.1107/S1600536810045502/hb5726sup1.cif

Structure factors: contains datablocks I. DOI: 10.1107/S1600536810045502/hb5726Isup2.hkl

Additional supplementary materials:  crystallographic information; 3D view; checkCIF report

## Figures and Tables

**Table 1:** Hydrogen-bond geometry (Å, °)

*D*—H⋯*A*	*D*—H	H⋯*A*	*D*⋯*A*	*D*—H⋯*A*
N2—H2⋯Cl2^i^	0.89 (1)	2.85 (1)	3.7228 (16)	167 (2)
O2—H2*A*⋯O1^ii^	0.82	1.97	2.7624 (19)	162

Symmetry codes: (i) 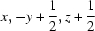; (ii) 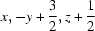.

**Fig. 1.**
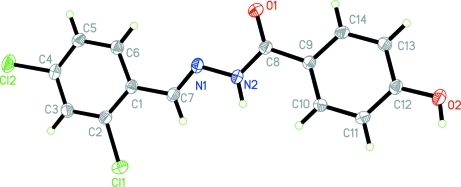
Molecular structure of the title compound, with 30% ellipsoids.

**Fig. 2.**
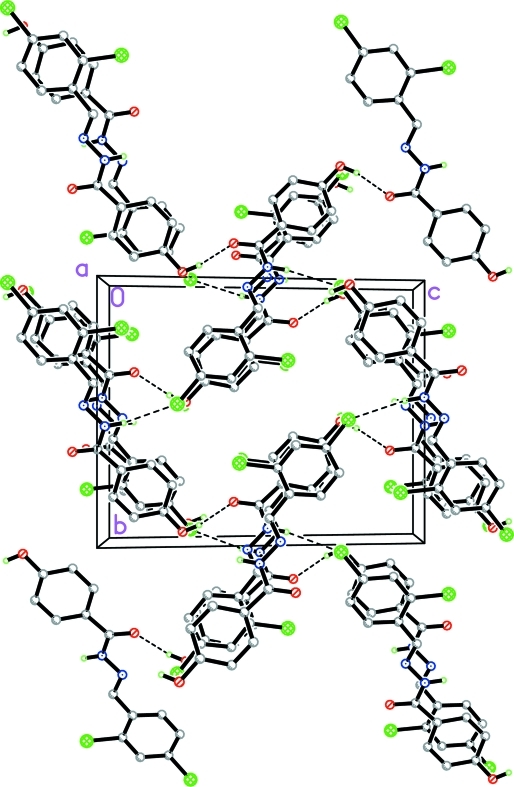
The molecular packing of the title compound, viewed along the a axis. Hydrogen bonds are drawn as thin dashed lines.

## supplementary crystallographic information

### Comment

The hydrazone compounds bearing –CH=N—NH—C(O)- groups have been received much attention for their structures (Carvalho *et al.*, 2010; Liu, 2010; Fun *et al.*, 2008; Wang *et al.*, 2010) and properties (Singh *et al.*, 2009; Zhu *et al.*, 2009; Vijayakumar *et al.*, 2009; Tameem *et al.*, 2010). In this paper, the title new hydrazone compound is reported.

The molecular structure of the title compound is shown in Fig. 1. The molecule adopts an *E* geometry with respect to the azomethine group. The dihedral angle between the two aromatic rings C1—C6 and C9—C14 is 7.0 (2)°. In the crystal structure, molecules are linked through intermolecular N–H···Cl and O–H···O hydrogen bonds to form a three-dimensional network (Table 1, Fig. 2).

### Experimental

Equimolar quantities (0.1 mmol each) of 2,4-dichlorobenzaldehyde and 4-hydroxybenzohydrazide were mixed and stirred in methanol for 30 min at reflux. After keeping the filtrate in air for a few days, colorless blocks of the title compound were formed.

### Refinement

H2 attached to N2 was located from a difference Fourier map, and refined with N–H distance restrained to 0.90 (1) Å, and with *U*_iso_ restrained to 0.08 Å^2^. The remaining H atoms were placed in calculated positions and constrained to ride on their parent atoms, with C—H distances of 0.93 Å, O—H distance of 0.85 Å, and with *U*_iso_(H) = 1.2*U*_eq_(C) and 1.5*U*_eq_(O).

### Crystal data

**Table:**

C_14_H_10_Cl_2_N_2_O_2_	*F*(000) = 632
*M**_r_* = 309.14	*D*_x_ = 1.529 Mg m^−^^3^
Monoclinic, *P*2_1_/*c*	Mo *K*α radiation, λ = 0.71073 Å
Hall symbol: -P 2ybc	Cell parameters from 3439 reflections
*a* = 7.6687 (11) Å	θ = 2.2–28.1°
*b* = 11.9591 (17) Å	µ = 0.49 mm^−^^1^
*c* = 15.043 (2) Å	*T* = 298 K
β = 103.200 (2)°	Block, colorless
*V* = 1343.2 (3) Å^3^	0.17 × 0.13 × 0.13 mm
*Z* = 4	

### Data collection

**Table:**

Bruker SMART CCD diffractometer	2838 independent reflections
Radiation source: fine-focus sealed tube	2385 reflections with *I* > 2σ(*I*)
graphite	*R*_int_ = 0.019
ω scans	θ_max_ = 27.0°, θ_min_ = 2.2°
Absorption correction: multi-scan (*SADABS*; Sheldrick, 1996)	*h* = −9→9
*T*_min_ = 0.922, *T*_max_ = 0.940	*k* = −7→15
6784 measured reflections	*l* = −19→17

### Refinement

**Table:**

Refinement on *F*^2^	Primary atom site location: structure-invariant direct methods
Least-squares matrix: full	Secondary atom site location: difference Fourier map
*R*[*F*^2^ > 2σ(*F*^2^)] = 0.041	Hydrogen site location: inferred from neighbouring sites
*wR*(*F*^2^) = 0.114	H atoms treated by a mixture of independent and constrained refinement
*S* = 1.05	*w* = 1/[σ^2^(*F*_o_^2^) + (0.0636*P*)^2^ + 0.4165*P*] where *P* = (*F*_o_^2^ + 2*F*_c_^2^)/3
2838 reflections	(Δ/σ)_max_ < 0.001
185 parameters	Δρ_max_ = 0.37 e Å^−^^3^
1 restraint	Δρ_min_ = −0.58 e Å^−^^3^

### Special details

**Table:**

Geometry. All e.s.d.'s (except the e.s.d. in the dihedral angle between two l.s. planes) are estimated using the full covariance matrix. The cell e.s.d.'s are taken into account individually in the estimation of e.s.d.'s in distances, angles and torsion angles; correlations between e.s.d.'s in cell parameters are only used when they are defined by crystal symmetry. An approximate (isotropic) treatment of cell e.s.d.'s is used for estimating e.s.d.'s involving l.s. planes.
Refinement. Refinement of *F*^2^ against ALL reflections. The weighted *R*-factor *wR* and goodness of fit *S* are based on *F*^2^, conventional *R*-factors *R* are based on *F*, with *F* set to zero for negative *F*^2^. The threshold expression of *F*^2^ > σ(*F*^2^) is used only for calculating *R*-factors(gt) *etc*. and is not relevant to the choice of reflections for refinement. *R*-factors based on *F*^2^ are statistically about twice as large as those based on *F*, and *R*- factors based on ALL data will be even larger.

### Fractional atomic coordinates and isotropic or equivalent isotropic displacement parameters (Å^2^)

**Table:**

	*x*	*y*	*z*	*U*_iso_*/*U*_eq_	
Cl1	1.05089 (9)	0.19236 (5)	0.07396 (3)	0.0590 (2)	
Cl2	0.99147 (8)	0.02938 (5)	−0.26115 (4)	0.05604 (19)	
N1	0.7367 (2)	0.47578 (12)	−0.05039 (10)	0.0350 (3)	
N2	0.7130 (2)	0.55434 (12)	0.01223 (10)	0.0363 (3)	
O1	0.5166 (2)	0.65200 (12)	−0.09421 (8)	0.0476 (4)	
O2	0.5103 (2)	0.96168 (11)	0.24525 (9)	0.0472 (4)	
H2A	0.5329	0.9360	0.2971	0.071*	
C1	0.8721 (2)	0.30387 (14)	−0.07778 (12)	0.0322 (4)	
C2	0.9708 (2)	0.20915 (15)	−0.04294 (12)	0.0360 (4)	
C3	1.0075 (2)	0.12484 (15)	−0.09862 (12)	0.0388 (4)	
H3	1.0745	0.0628	−0.0741	0.047*	
C4	0.9423 (2)	0.13500 (15)	−0.19144 (12)	0.0372 (4)	
C5	0.8419 (2)	0.22625 (15)	−0.22950 (12)	0.0385 (4)	
H5	0.7984	0.2313	−0.2924	0.046*	
C6	0.8074 (2)	0.30948 (15)	−0.17268 (12)	0.0351 (4)	
H6	0.7395	0.3709	−0.1979	0.042*	
C7	0.8350 (2)	0.39312 (15)	−0.01809 (12)	0.0365 (4)	
H7	0.8840	0.3892	0.0444	0.044*	
C8	0.5992 (2)	0.64163 (14)	−0.01442 (11)	0.0325 (4)	
C9	0.5824 (2)	0.72236 (13)	0.05731 (11)	0.0307 (4)	
C10	0.6378 (2)	0.70117 (15)	0.15075 (12)	0.0358 (4)	
H10	0.6907	0.6329	0.1704	0.043*	
C11	0.6154 (2)	0.77948 (15)	0.21432 (11)	0.0375 (4)	
H11	0.6530	0.7637	0.2763	0.045*	
C12	0.5367 (2)	0.88225 (14)	0.18611 (11)	0.0338 (4)	
C13	0.4802 (3)	0.90445 (15)	0.09322 (12)	0.0391 (4)	
H13	0.4276	0.9729	0.0736	0.047*	
C14	0.5020 (3)	0.82533 (14)	0.03025 (11)	0.0370 (4)	
H14	0.4623	0.8408	−0.0317	0.044*	
H2	0.767 (3)	0.542 (2)	0.0707 (8)	0.080*	

### Atomic displacement parameters (Å^2^)

**Table:**

	*U*^11^	*U*^22^	*U*^33^	*U*^12^	*U*^13^	*U*^23^
Cl1	0.0820 (4)	0.0559 (3)	0.0333 (3)	0.0123 (3)	0.0010 (2)	−0.0002 (2)
Cl2	0.0620 (3)	0.0532 (3)	0.0521 (3)	0.0072 (2)	0.0113 (2)	−0.0228 (2)
N1	0.0433 (8)	0.0303 (7)	0.0336 (7)	−0.0054 (6)	0.0135 (6)	−0.0062 (6)
N2	0.0461 (8)	0.0315 (7)	0.0307 (7)	0.0008 (6)	0.0074 (6)	−0.0064 (6)
O1	0.0696 (10)	0.0455 (8)	0.0266 (6)	0.0091 (7)	0.0087 (6)	−0.0004 (5)
O2	0.0715 (9)	0.0374 (7)	0.0341 (7)	0.0030 (6)	0.0149 (7)	−0.0066 (5)
C1	0.0344 (9)	0.0301 (8)	0.0339 (8)	−0.0059 (6)	0.0117 (7)	−0.0039 (7)
C2	0.0391 (9)	0.0356 (9)	0.0327 (8)	−0.0034 (7)	0.0071 (7)	−0.0019 (7)
C3	0.0398 (10)	0.0332 (9)	0.0426 (10)	0.0007 (7)	0.0077 (8)	−0.0033 (7)
C4	0.0367 (9)	0.0364 (9)	0.0406 (9)	−0.0051 (7)	0.0132 (7)	−0.0114 (7)
C5	0.0429 (10)	0.0425 (10)	0.0308 (8)	−0.0025 (8)	0.0098 (7)	−0.0034 (7)
C6	0.0377 (9)	0.0326 (9)	0.0360 (9)	−0.0027 (7)	0.0106 (7)	0.0006 (7)
C7	0.0433 (10)	0.0348 (9)	0.0321 (8)	−0.0041 (7)	0.0097 (7)	−0.0045 (7)
C8	0.0410 (9)	0.0302 (8)	0.0279 (8)	−0.0062 (7)	0.0111 (7)	0.0000 (6)
C9	0.0356 (9)	0.0285 (8)	0.0291 (8)	−0.0048 (6)	0.0098 (6)	−0.0003 (6)
C10	0.0418 (10)	0.0357 (9)	0.0299 (8)	0.0070 (7)	0.0082 (7)	0.0037 (7)
C11	0.0443 (10)	0.0422 (10)	0.0251 (8)	0.0050 (8)	0.0060 (7)	0.0015 (7)
C12	0.0416 (9)	0.0304 (8)	0.0314 (8)	−0.0062 (7)	0.0124 (7)	−0.0032 (7)
C13	0.0576 (11)	0.0261 (8)	0.0342 (9)	0.0001 (8)	0.0117 (8)	0.0045 (7)
C14	0.0528 (11)	0.0319 (9)	0.0259 (8)	−0.0026 (7)	0.0082 (7)	0.0037 (6)

### Geometric parameters (Å, °)

**Table:**

Cl1—C2	1.7369 (18)	C5—C6	1.377 (2)
Cl2—C4	1.7372 (18)	C5—H5	0.9300
N1—C7	1.270 (2)	C6—H6	0.9300
N1—N2	1.372 (2)	C7—H7	0.9300
N2—C8	1.361 (2)	C8—C9	1.475 (2)
N2—H2	0.894 (10)	C9—C10	1.396 (2)
O1—C8	1.229 (2)	C9—C14	1.396 (2)
O2—C12	1.348 (2)	C10—C11	1.377 (2)
O2—H2A	0.8200	C10—H10	0.9300
C1—C2	1.396 (2)	C11—C12	1.392 (2)
C1—C6	1.402 (2)	C11—H11	0.9300
C1—C7	1.464 (2)	C12—C13	1.391 (2)
C2—C3	1.380 (2)	C13—C14	1.376 (2)
C3—C4	1.377 (3)	C13—H13	0.9300
C3—H3	0.9300	C14—H14	0.9300
C4—C5	1.382 (3)		
			
C7—N1—N2	115.49 (14)	N1—C7—H7	119.5
C8—N2—N1	119.98 (14)	C1—C7—H7	119.5
C8—N2—H2	122.6 (18)	O1—C8—N2	121.20 (16)
N1—N2—H2	117.1 (18)	O1—C8—C9	122.47 (16)
C12—O2—H2A	109.5	N2—C8—C9	116.33 (14)
C2—C1—C6	117.17 (16)	C10—C9—C14	117.80 (15)
C2—C1—C7	121.73 (15)	C10—C9—C8	124.10 (15)
C6—C1—C7	121.09 (16)	C14—C9—C8	118.08 (14)
C3—C2—C1	122.21 (16)	C11—C10—C9	121.24 (16)
C3—C2—Cl1	117.13 (14)	C11—C10—H10	119.4
C1—C2—Cl1	120.66 (14)	C9—C10—H10	119.4
C4—C3—C2	118.28 (17)	C10—C11—C12	120.19 (15)
C4—C3—H3	120.9	C10—C11—H11	119.9
C2—C3—H3	120.9	C12—C11—H11	119.9
C3—C4—C5	121.92 (16)	O2—C12—C13	117.99 (16)
C3—C4—Cl2	117.99 (14)	O2—C12—C11	122.74 (15)
C5—C4—Cl2	120.08 (14)	C13—C12—C11	119.26 (16)
C6—C5—C4	118.79 (16)	C14—C13—C12	120.10 (16)
C6—C5—H5	120.6	C14—C13—H13	120.0
C4—C5—H5	120.6	C12—C13—H13	120.0
C5—C6—C1	121.61 (17)	C13—C14—C9	121.41 (15)
C5—C6—H6	119.2	C13—C14—H14	119.3
C1—C6—H6	119.2	C9—C14—H14	119.3
N1—C7—C1	120.95 (16)		

### Hydrogen-bond geometry (Å, °)

**Table:**

*D*—H···*A*	*D*—H	H···*A*	*D*···*A*	*D*—H···*A*
N2—H2···Cl2^i^	0.89 (1)	2.85 (1)	3.7228 (16)	167 (2)
O2—H2A···O1^ii^	0.82	1.97	2.7624 (19)	162

Symmetry codes: (i) *x*, −*y*+1/2, *z*+1/2; (ii) *x*, −*y*+3/2, *z*+1/2.
